# Expression of Insl3 Protein in Adult *Danio rerio*

**DOI:** 10.3390/ijms25105419

**Published:** 2024-05-16

**Authors:** Aldo Donizetti, Mauro Calicchio, Maria Zelinda Romano, Luigi Rosati, Manuela Turco, Anna Maria Carrese, Rosanna del Gaudio, Ida Ferrandino, Francesco Aniello

**Affiliations:** 1 Department of Biology, University of Naples Federico II, 80126 Naples, Italy; aldo.donizetti@unina.it (A.D.); m.calicchio@studenti.unina.it (M.C.); luigi.rosati@unina.it (L.R.); turcomanuela02@gmail.com (M.T.); annamaria.carrese@unina.it (A.M.C.); rdelgaud@unina.it (R.d.G.); ida.ferrandino@unina.it (I.F.); 2 Dipartimento di Medicina Sperimentale, Università degli Studi della Campania “Luigi Vanvitelli”, 80138 Naples, Italy; mariazelinda.romano@unicampania.it

**Keywords:** Insl3, Rxfp2, *Dano rerio*, testis, antibody insulin-like peptide C, immunohistochemistry, reproduction

## Abstract

Insulin-like peptide 3 (INSL3) is a biomarker for Leydig cells in the testes of vertebrates, and it is principally involved in spermatogenesis through specific binding with the RXFP2 receptor. This study reports the *insl3* gene transcript and the Insl3 prepropeptide expression in both non-reproductive and reproductive tissues of *Danio rerio*. An immunohistochemistry analysis shows that the hormone is present at a low level in the Leydig cells and germ cells at all stages of *Danio rerio* testis differentiation. Considering that the *insl3* gene is transcribed in Leydig cells, our results highlight an autocrine and paracrine function of this hormone in the *Danio rerio* testis, adding new information on the Insl3 mode of action in reproduction. We also show that Insl3 and Rxfp2 belonging to *Danio rerio* and other vertebrate species share most of the amino acid residues involved in the ligand–receptor interaction and activation, suggesting a conserved mechanism of action during vertebrate evolution.

## 1. Introduction

INSL3 is one of the members of the insulin/relaxin family, the evolutionary origin of which has been extensively analyzed in the vertebrate lineage [1,2,3]. In humans, the members of the insulin-like/relaxins family are encoded by seven genes: *RLN1*, *RLN2*, *RLN3*, and the insulin-like peptides (INSL) *INSL3*, *INSL4*, *INSL5*, and *INSL6* [4]. All these relaxin family peptides act on a group of four G protein-coupled receptors, RXFP1, RXFP2, RXFP3, and RXFP4, which are the physiological targets of the relaxin/insulin-like peptide family [5]. Among these four receptors, RXFP2 is the physiological ligand for the INSL3 peptide [6,7]. Adham and colleagues [8] were the first to identify the *insl3* transcript in the Leydig cell (LC) from a boar testis cDNA library. Later, the *insl3* transcript was also found in the LC of mouse testis [9] and in the LC of human testis, suggesting that INSL3 is an important hormone produced by the LC in humans, serving as a specific biomarker for this cell type [10]. The presence of Insl3 and the specific receptor Rxfp2 in the testes was also studied in boars. While *insl3* was identified in the LC, *Rxfp2* expression was mainly found in meiotic and post-meiotic germ cells but not in the LC [11]. Also, in female goats, *insl3* transcription is present in the corpus luteum, while its specific receptor, Rxfp2, is found in cell types of the intra- and extra-ovarian reproductive organs, demonstrating that the hormone-receptor system may operate during pregnancy [12], along with the early role of Insl3 evidenced in the LC of boar testis [13]. In the mouse, *insl3* RNA and protein are present during testicular and ovarian tissue development and differentiation [14]. Mice mutants for *insl3* are viable but exhibit bilateral cryptorchidism caused by abnormalities in gubernaculum development, testis, and genital ducts, leading to abnormal spermatogenesis and infertility [15,16]. A Western blot analysis on blood reveals that Insl3, secreted by the testicular LC, is a monomeric protein comprising three B–C–A domains with full biological activity in boars [17]. It is known that the mature form of Insl3 consists of a small peptide (about 6000 Dalton in size) that possesses the heterodimeric insulin-type A-B structure and is the physiological ligand of relaxin family peptide receptor 2 (RXFP2) [18]. Up to now, the *insl3* gene has been identified in many vertebrates from fish to primates, and the transcript is mainly expressed in the LC, confirming a key role in all organisms, perhaps with different functions, but mostly involved in spermatogenesis and pregnancy. In addition, the *INSL3* gene in humans is also expressed in many other organs, such as the ovary, testis, and others [19]. The majority of *insl3* gene expression studies have been conducted in the mammalian LC, and other studies have been carried out using other vertebrates, such as amphibians and teleosts. It is noteworthy to mention that mRNA expression of the amphibian *insl3*, termed (fRLX), has been evidenced in the testis of the frog *Rana esculenta*, specifically in the interstitial LC by in situ hybridization [20], and its expression is regulated by testosterone [21]. Also, in *Danio rerio* testis, *insl3* mRNA expression was evidenced by in situ hybridization in the LC [22], as well as by RT qPCR in the ovary, brain, and gill [1]. In addition, it has been reported that Insl3 recruits two locally active receptors Rxfp2a and Rxfp2b in testis tissue for spermatogenesis, and the hormone (Fsh) increases the production of insulin-like 3 [23]. Three *rxfp2* genes (*rxfp2*a, *rxfp2*b, and *rxfp2l*) are present in the *Danio rerio*. The transcripts of all three genes are maternally derived and present in fertilized eggs; later, during development, *rxfp2*a is only expressed at the larval stage, whereas *rxfp2*b and *rxfp2l* are expressed in all the analyzed stages, indicating a role for this receptor and Insl3 in vertebrate embryonic development [24].

In this work, we demonstrate the expression of *insl3* in both reproductive and non-reproductive tissues. We show that the prohormone is present at a low level in the Leydig and germ cells at all stages of differentiation by using an antibody against the C peptide in immunohistochemistry assays. Our data led us to hypothesize that Insl3 prohormone is synthesized in the LC and acts in a paracrine and autocrine fashion on the different cell types of *Danio rerio* testis. In addition, we evaluated the conservation of amino acids involved in the ligand–receptor interaction and showed that for the vast majority they are shared in the analyzed species. This highlights the need to evaluate the mechanism of action proposed in this study in other species as well.

## 2. Results

### 2.1. Expressions of Insl3 Gene

**Figure 1:**
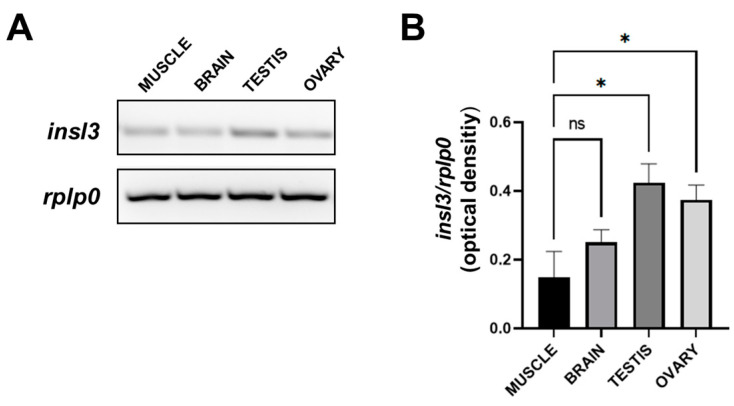
Gene expression pattern for *insl3* gene by RT-PCR analysis. (**A**) RT-PCR gel image. (**B**) Relative statistical analysis of *insl3* expression in zebrafish tissues. Data were normalized with *rplp0* cDNA and reported as OD ratio. All values are expressed as means ± SEM. *: *p* ≤ 0.05; ns: not significant.

We evaluated the expression of the *insl3* gene by the analysis of mRNA in various tissues. In particular, the level of the transcript was analyzed in the skeletal muscle, brain, testis, and ovary. As shown in Figure 1A, *insl3* mRNA is present in all tissues, with the lowest level in the muscle and the brain and the highest levels in the reproductive organs. The expression levels are displayed in the graph, which shows a higher expression level in the testis compared to the ovary, followed by the brain and muscle (Figure 1B). These results show that gene transcription of *insl3* is not limited to the testis and ovary but also in different tissues as also reported in [1].

### 2.2. Insl3 Protein Level in Danio rerio Tissues

**Figure 2:**
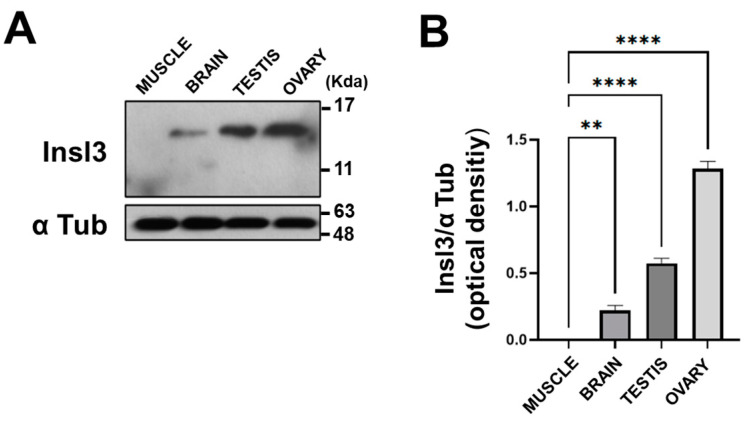
Protein expression pattern of Insl3 by Western blotting analysis. (**A**) Western blot and (**B**) relative statistical analysis of Insl3 protein levels in *Danio rerio* tissues. Data were normalized with α-Tubulin and reported as OD ratio. All values are expressed as means ± SEM. **: *p* ≤ 0.01; ****: *p* ≤ 0.0001.

To analyze the protein expression, we performed a Western blot analysis using an antibody against an Insl3 protein region corresponding to the C peptide. No signal was revealed for the muscle tissue, while the protein was found in the brain, testis, and ovary (Figure 2A). The expression levels are displayed in the graph (Figure 2B), which shows a higher expression level in the ovary compared to the testis and brain.

### 2.3. Insl3 Protein Localization in Danio rerio Testis

**Figure 3:**
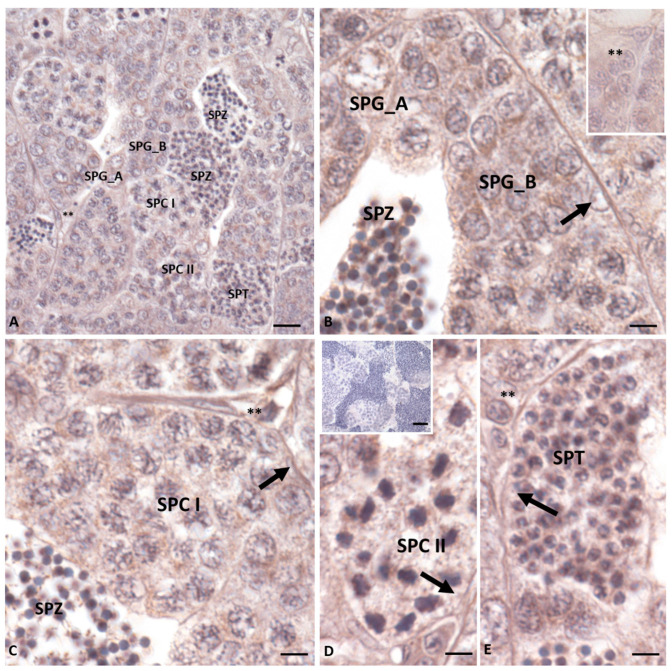
Immunohistochemistry for Insl3 in *Danio rerio* testis. The immunolocalization signal appears as brown areas. (**A**–**E**): Antibody positivity is evident in Spermatogonia A (SPG_A), Spermatogonia B (SPG_B), Spermatocytes I (SPC I), Spermatocytes II (SPC II), and Spermatids (SPT) and Spermatozoa (SPZ). The low signal is evident in the LC (asterisks) and no positivity is found in the Sertoli cells (arrow). The negative control sections ((**D**) inset) show no signal. The scale bars correspond to 50 µm in the figure (**D**) insert, 20 µm in figure (**A**), and 10 µm in figures (**B**–**E**).

We used the same antibody for the immunohistochemistry experiments on the testis sections. The analysis revealed a low signal in the LC (Figure 3A–C,E); likewise, the Sertoli cells within the cysts were not stained regardless of the stage of germ cell differentiation (Figure 3B–E). In contrast, it was possible to highlight a strong signal in the germ cells at all stages of differentiation as Spermatogonia A and B, Spermatocytes I and II, and Spermatids and Spermatozoa (Figure 3A–E). The Figure 3D inset shows the negative controls, obtained by omission of the primary antibody.

### 2.4. Comparison of Danio rerio Insl3 and Rxfp2 Protein Sequence with Other Vertebrate Species

**Figure 4:**
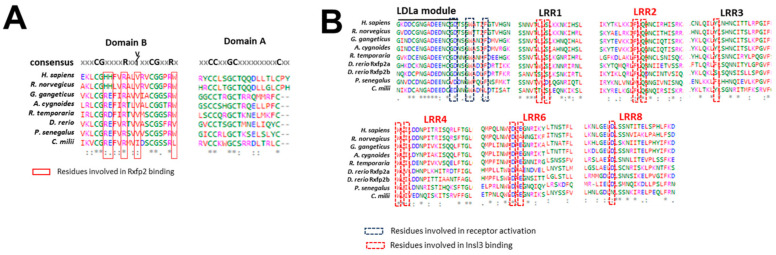
Analysis of Insl3 and Rxfp2 sequences in vertebrate species. (**A**) Sequence alignment of the Insl3 protein *Homo sapiens* Insl3 (NP_005534.2); *Rattus norvegicus* Insl3 (NP_446132.1); *Gavialis gangeticus* Insl3 (XP_019379679.1); *Anser cygnoides* Insl3 (XP_047908400.1); *Rana temporaria* Insl3 (XP_040177352.1); *Danio rerio* Insl3 (NP_001108525.2); *Polypterus senegalus* Insl3 (XP_039623679.1); and *Callorhinchus milii* Insl3 (AFP04465.1). (**B**) Sequence alignment of the Rxfp2 extracellular domain. *Homo sapiens* (NP_570718.1); *Rattus norvegicus* (NP_001012493.1); *Gavialis gangeticus* (XP_019381047.1); *Anser cygnoides* (XP_013044814.2); *Rana temporaria* (XP_040196386.1); *Danio rerio* Rxfp2b (NP_001315011.1); *Danio rerio* Rxfp2a (NP_001315313.1); *Polypterus senegalus* (XP_039622298.1); and *Callorhinchus milii* (XM_00789132.1). Identical amino acids are indicated by asterisks; conservative substitutions are shown by colons and semiconservative substitutions by dots. Different colors represent the physiochemical properties of amino acids.

In addition to the *Danio rerio* sequences, we retrieved their amino acid sequences from at least one species for any vertebrate class. The entire Insl3 sequence alignment is reported in Figure S1A. The percentage of the sequence identity ranged from 59% of *Rattus norvegicus* to 21% of *Callorhinchus milii* when compared to the human sequence (Figure S1A). As expected, the highest identity is related to the B and A peptides that form the mature protein, whereas the C peptide was the most variable part of the precursor. Domain B and A presented a consensus sequence conserved in all the analyzed species; this sequence included the cysteine residues involved in the disulfide bond and two residues involved in the receptor interaction (Figure 4A). The residues involved in the receptor interaction have been identified in human sequences [25,26] and are evidenced in the red squares of Figure 4A. The two histidine residues present in human and rat Insl3 sequences are replaced with the most similar arginine for the first one and by glutamic and aspartic acid for the second one in the other analyzed species (Figure 4A). The arginine is conserved in all the sequences; in contrast, the valine is replaced with isoleucine in the *Callorhinchus milii* species (Figure 4A). Finally, the highest variability is found for the tryptophan residue that is changed in valine in the *Danio rerio* sequence and leucine in the *Callorhinchus milii* sequence (Figure 4A).

Rxfp2 is a G-protein receptor with an N-terminal (LDLa) module, a linker region and a large extracellular domain consisting of 10 leucine-rich repeats (LRRs) [27]. Regarding Rxfp2, the percentage of identity ranged from 82.61% of *Rattus norvegicus* to 58.81% of *Callorhinchus milii* when compared to the human protein sequence (Figure S1B). Relevant amino acid residues for the human receptor activation are confined between the LDLa module and the linker sequence and consist of the GD**GW***F sequence [28,29,30]. This consensus sequence is conserved in all the analyzed vertebrate sequences with a variation occurring for glycine residue that is changed with a valine or glutamic acid in the ancient fishes (Figure 4B). Amino acids involved in the interaction with INSL3 in human are distributed in LRR1, 2, 3, 4, 6, and 8 [25,26]. In total, 6 out of 10 amino acids are conserved in the analyzed species, whereas the other 4 are variable in fish species (Figure 4B). The leucin in the LRR1 region is substituted with serine in the *Danio rerio* Rxfp2b sequence and with threonine in the *Callorhinchus milii* sequence (Figure 4B). The serine in the LRR1 region is substituted with aspartic acid in *Polypterus senegalus* (Figure 4B). The tyrosine in LRR3 is substituted with phenylalanine in *Danio rerio* Rxfp2a and the *Polypterus senegalus* sequence (Figure 4B). Finally, the aspartic acid in LRR6 is substituted with alanine in the *Rattus norvegicus* and *Danio rerio* Rxfp2a sequence (Figure 4B).

## 3. Discussion

INSL3 is a peptide hormone that, through interaction with the RXFP2 receptor, causes an increase in cAMP production [7,25] and performs several important physiological roles in humans, including reproduction and cardiovascular function. It has been reported that INSL3 is a major product of the testicular LC in adults, regulating the spermatogenesis process [31]. Notably, the circulating INSL3 hormone is functionally relevant in human males [32,33]. In humans, INSL3 is extensively expressed in the testis; in contrast, the cognate receptor is expressed in many other tissues, including at high levels in the adrenal gland, endometrium, and lung and medium levels in the brain, heart, ovary, and spleen [19]. The expression pattern of the receptor reflects the physiologic functions of INSL3 not only in reproduction but also in different tissues/organs [34].

Analyses by RT-PCR showed the expression of the *insl3* transcript in all the analyzed tissues of *Danio rerio*, with the highest levels for the reproductive organs. The localization of the *insl3* mRNA was demonstrated in the *Danio rerio* testis by in situ hybridization [22], while Alnafea and colleagues [35] reported, by RT-PCR, the expression in the development embryo from 1 to 6 days in *Oryzias latipes*. On the other hand, the Western blotting analysis showed no signal for muscle tissue, while the protein was revealed in the brain and the ovary and testis with the highest levels. Also, the Western blot shows proteins are present in various tissues, indicating that the testis is not the exclusive site of Insl3 production. To add further details on the expression of the protein in the same tissue, we used the same antibody by the immunohistochemistry approach. Crespo and colleagues [23] reported the localization of the receptors Rxfp2a and 2b in the type A Spermatogonia and Sertoli cells in the testis. We did not detect immunohistochemistry signals in the Sertoli cells perhaps due to the low level of the hormone, while we revealed the presence of Insl3 in Spermatogonia A, Spermatogonia B, Spermatocytes I, Spermatocytes II, Spermatids, Spermatozoa and at a low level in the LC. Our analysis suggests that regardless of the cells that produce Insl3 (presumably the LC), the prohormone is also found in other types of cells where it would exert its function in a paracrine and autocrine fashion.

With the idea of providing an update on the conservation of the INSL3/RXFP2 pair in vertebrates, we retrieved amino acid sequences from species belonging to mammals, birds, reptiles, amphibians, ray-finned fishes (teleost and non-teleost fishes), and cartilaginous fishes. The novelty of this work was the finding of the Insl3 sequence in birds, reptiles, and cartilaginous fishes that were missing in previously published evolutionary studies, probably due to a lower number of genomic sequences related to those vertebrate classes. The amino acid sequence alignment provided the identification of a consensus sequence in the B domain (CG****R**V**CG***R) and the A domain (CC**GC*********C) present in all the analyzed vertebrate species. Overall, the alignment confirmed that the most conserved regions are the B and A peptides, while the C peptide showed the highest variability. The amino acid residues of human INSL3 involved in the receptor interaction have been identified and include His (B12), His (B13), Arg (B16) and Val (B19), Arg (B20), and Trp (B27) [25,26]. Our analyses showed that two amino acids are particularly divergent. The histidine B13 of mammals is substituted by negatively charged aspartic or glutamic acid, and tryptophan B27 is changed in valine (*Danio rerio*) and leucine (*Callorhinchus milii*). Of particular interest are the two histidine residues involved in the receptor interaction that are perfectly conserved in rats and humans, while they are substituted by arginine and by glutamic or aspartic acid in the other classes, from cartilaginous fishes to birds. The amino acid alignment highlighted that, similarly to the INSL3 ligand, most receptor residues involved in the activation and binding are conserved, while others are different, likely because of ligand–receptor co-evolution as also hypothesized by Good and colleagues [2].

## 4. Materials and Methods

### 4.1. Bioinformatics Analysis

We retrieved the amino acid sequence of Insl3 and Rxfp2 from the NCBI database by using the gene symbol and accession number when available or by tBLASTn search using the amino acid sequence as a bait. The alignments were performed by Clustal Omega version 1.2.4 with the default parameters [36,37].

### 4.2. Animals

The adult zebrafish were at the Department of Biology of the University of Naples Federico II and housed in tanks with a photoperiod of 12:12 h light/dark, at a temperature of 28 °C. The fish were fed twice a day with a commercial diet supplemented with *Artemia* sp. *nauplii* essentially as reported in Fiengo et al. [38]. The fish were all euthanized with ethyl 3-aminobenzoate methane sulfonate (MS-222, Sigma Aldrich^®^, Munich, Germany) before being sacrificed.

### 4.3. Expression of Danio rerio Insl3 mRNA in Tissues

Expression of the *insl3* gene was analyzed by the RT–PCR amplification method as already reported in Donizetti et al., 2015 [39]. Briefly, the first strand of cDNA was obtained from RNA samples of the muscle, brain, testis, and ovary. The total RNA from the adult tissues was isolated using the TRIgidy G reagent (AppliChem^®^, Darmstadt, Germany) following the manufacturer’s instructions. A total of 1 µg of RNA was retrotranscribed by the LunaScript RT SuperMix Kit (BioLabs^®^, Ipswich, MA, USA) in a final volume of 20 µL and following the manufacturer’s instructions. At the end of the reaction, the volume was brought to 50 µL by adding 30 µL of sterile water. For RT–PCR amplification, the following primers were used: insl3 (NM_001115053.2), forward (5′-ACTTCGCATACCCTTATAGGAATC-3′), and reverse (5′-CTCTGGTGCACAACGAGGTC-3′). The RT–PCR sensitivity was monitored on the cDNA of the ribosomal protein Rplp0 (NM_131580) with the following primers: forward (5′-CTGGAAAACAACCCAGCTCT-3′) and reverse (5′-CGGACCTCAGTCAGATCCTC-3′). In total, 2 µL of cDNA (40 ng) was used for the PCR reactions. The PCR reactions were carried out in a GeneAmp PCR System 9700 (Applied Biosystems) and consisted of an initial step at 95 °C for 5 min, followed by 36 cycles at 95 °C for 30 s, 58 °C for 40 s, and 72 °C for 1 min and a final cycle of extension at 72 °C for 5 min.

### 4.4. Production of Antibody

The polyclonal antisera were raised in rabbits using a synthetic peptide for immunization. The peptide INSL3 peptide C with the following sequence RDTPESVRGHPDPR was synthesized by an external service. After cross-linking with albumin by formaldehyde treatment, following the protocol reported in Donizetti et al., 2023 [40], the specificity of the INSL3 antiserum was checked by pre-adsorbing the primary antiserum with a five-fold excess of the corresponding epitope and assessed via Western blotting analysis and immunohistochemistry.

### 4.5. Western Blotting (WB) Analysis

The proteins were extracted from the muscle, brain, testis, and ovary in RIPA lysis buffer (TCL131; HIMedia^®^ Laboratories GmbH, Homburg, Germany) supplemented with a protease inhibitor mix (39102.01; SERVA Electrophoresis GmbH, Heidelberg, Germany). The samples were sonicated three times (20 Hz for 20 s each), placed on ice for 30 min, and then centrifuged at 10,000× *g* for 30 min at 4 °C. The supernatants were collected. Forty micrograms of the protein extracts was separated into SDS-PAGE (9–15% acrylamide) and treated according to Romano et al. [41]. Then, they were incubated overnight at 4 °C with primary antibodies as follows: anti-INSL3 (1:800) and anti-α-Tubulin (1:5000, E-AB-20036, Elabscience^®^, Houston, TX, USA). After the incubation, the filters were washed three times in TBST and incubated with peroxidase-conjugated secondary antibody anti-mouse IgG (1:5000, AP130P, Sigma Aldrich^®^, Munich, Germany) for the mouse and anti-α-Tubulin or anti-rabbit IgG (1:3000 AP307P; Sigma-Aldrich^®^, Munich, Germany) secondary antibody for the rabbit anti-INSL3 for 1 h at RT. Then, the filters were washed in TBST three times. The immunocomplexes were detected using the enhanced chemiluminescence (ECL) WB detection system. ImageJ software (version 1.53 g; NIH) was used to analyze all the bands.

### 4.6. Immunohistochemistry

Paraffin-embedded Blouin’s fixed testis were cut at 5 µm sections and used for the immunohistochemistry analysis, as previously reported [42]. Briefly, the slides were dewaxed and heat-treated in the microwave (2 × 10 min), using 0.1 M citrate buffer (pH 6.0) for antigen retrieval. After being washed in PBS, the sections were first rinsed with 2.5% H_2_O_2_ for 40 min to inactivate the endogenous peroxidases and then blocked for 1 h with normal goat serum (Pierce, Rockford, IL, USA) to reduce the non-specific background. Sections were incubated overnight at 4 °C with the primary antibody rabbit anti-INSL-3 and diluted 1:200 in normal goat serum, and this antibody has been previously validated by Western blotting. The day after, the reaction was revealed with a biotin-conjugated goat anti-rabbit secondary antibody (Kit Pierce, diluted 1:2000 in normal goat serum) and an avidin-biotin–peroxidase complex (ABC immunoperoxidase Kit, Pierce), using diaminobenzidine (DAB) as the chromogen. Sections were counterstained with Mayer’s hematoxylin. Negative controls were performed by omitting incubation with the primary antibody. The immunohistochemical signal was analyzed using a Zeiss Axioskop microscope and the images were acquired by using Axiovision 4.7 Software (Zeiss, Oberkochen, Germany).

### 4.7. Statistical Analysis

The results were obtained by analyzing the RNA and proteins extracted from three independent pools of organs. The data are expressed as mean ± SEM. A statistical analysis of the RT-PCR and WB data was carried out with one-way ANOVA using Prism 9.2.0, GraphPad Software (San Diego, CA, USA). The differences between the groups were considered statistically significant at *p* < 0.05.

## 5. Conclusions

The *Danio rerio insl3* gene transcript and prepropeptide expression are reported. Our results highlight a paracrine and autocrine function of this hormone in the *Danio rerio* testis and act as a propeptide, adding new information on the Insl3 mode of action in vertebrates. Using bioinformatic analyses, we characterized the INSL3 and RXFP2 proteins in vertebrates. We show that all the analyzed species shared most of the amino acid residues involved in the ligand–receptor interaction and activation.

## Data Availability

All data are available upon reasonable request to the corresponding author.

## Supplementary Materials

The following supporting information can be downloaded at: https://www.mdpi.com/article/10.3390/ijms25105419/s1.
