# Supplementary material for: Expression of Insl3 Protein in Adult Danio rerio
Source: Int J Mol Sci. 2024 May 16;25(10):5419. doi: 10.3390/ijms25105419 (PMC11122137; doi:10.3390/ijms25105419)

Supplementary materials

Legends

Figure S1: Sequence alignment of the entire Insl3 sequence of various vertebrate species as reported in Figure 1. In *D.rerio*, the part corresponding to peptide C used for antibody production is highlighted in red. The percentage value indicates the amino acid sequence identity of the full-length proteins.

Figure S2: Sequence alignment of the entire Rxfp2 sequence of various vertebrate species as reported in Figure 2. The percentage value indicates the amino acid sequence identity of the full-length proteins.

Figure S1

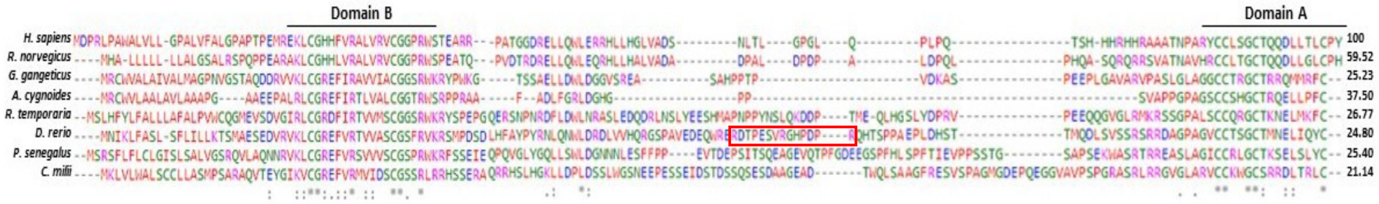

Figure S2

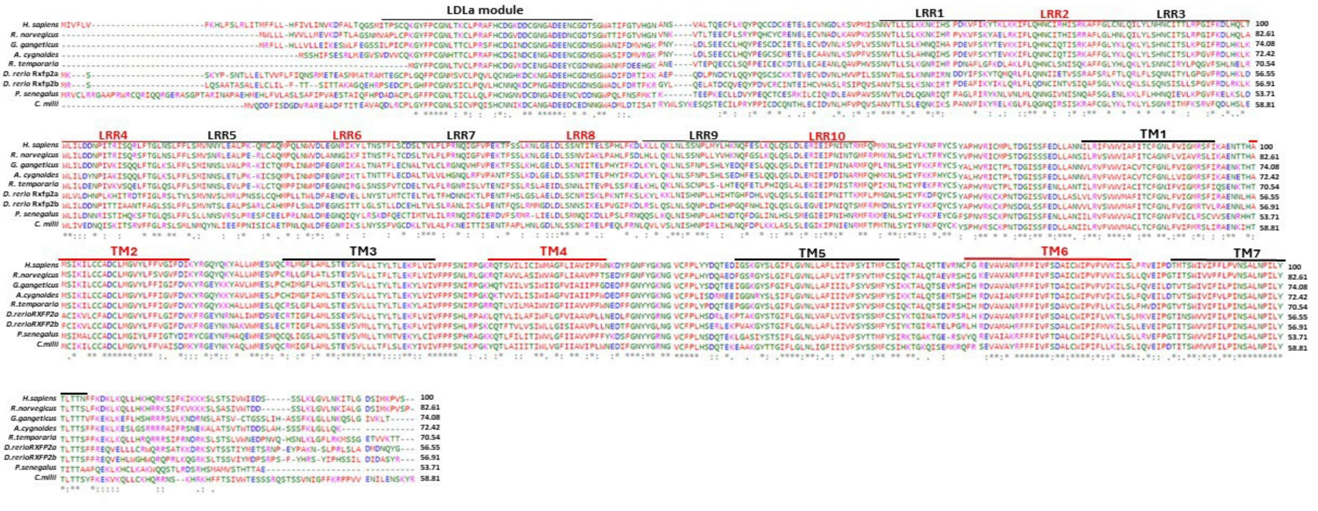

Supplement: Supplementary file 1 [file ijms-25-05419-s001.zip › ijms-2972691-supplementary.pdf]
